# The Dynamic Changes of Gut Microbiota in *Muc2* Deficient Mice

**DOI:** 10.3390/ijms19092809

**Published:** 2018-09-18

**Authors:** Minna Wu, Yaqi Wu, Jianmin Li, Yonghua Bao, Yongchen Guo, Wancai Yang

**Affiliations:** 1 College of Basic Medicine, Xinxiang Medical University, Xinxiang 453003, Henan, China; happy_minzi@163.com (M.W.); yaqiwu2018@126.com (Y.W.); jianminli1990@163.com (J.L.); 2 Department of Pathology, Institute of Precision Medicine, Jining Medical University, Jining 272067, Shandong, China; Baoyonghua2005@126.com (Y.B.); Guoyongchen2005@126.com (Y.G.); 3 Department of Pathology, University of Illinois at Chicago, Chicago, IL 60612, USA

**Keywords:** mucin, microbiota, colitis, carcinogenesis, colorectal cancer

## Abstract

Gut dysbiosis is associated with colitis-associated colorectal carcinogenesis, and the genetic deficiency of the *Muc2* gene causes spontaneous development of colitis and colorectal cancer. Whether there are changes of gut microbiota and a linkage between the changes of microbiota and intestinal pathology in *Muc2^−/−^* mice are unclear. *Muc2^−/−^* and *Muc2^+/+^* mice were generated by backcrossing from *Muc2^+/−^* mice, and the fecal samples were collected at different dates (48th, 98th, 118th, 138th, and 178th day). Gut microbiota were analyzed by high-throughput sequencing with the universal 16S rRNA primers (V3–V5 region). All mice were sacrificed at day 178 to collect colonic tissue and epithelial cells for the analysis of histopathology and inflammatory cytokines. On the 178th day, *Muc2^−/−^* mice developed colorectal chronic colitis, hyperplasia, adenomas and adenocarcinomas, and inflammatory cytokines (e.g., cyclooxygenase 2 (COX-2), interleukin 6 (IL-6), tumor necrosis factor-α (TNF-α), interleukin 1 β (IL-1β), i-kappa-B-kinase β (IKKβ)) were significantly increased in colonic epithelial cells of *Muc2^−/−^* mice. In general, structural segregation of gut microbiota was observed throughout the experimental time points between the *Muc2^−/−^* and *Muc2^+/+^* mice. Impressively, in *Muc2^−/−^* mice, Alpha diversities reflected by Shannon and Chao indexes were higher, the phylum of Firmicutes was enriched and Bacteroidetes was decreased, and *Desulfovibrio*, *Escherichia*, *Akkermansia*, *Turicibacter*, and *Erysipelotrichaceae* were significantly increased, but *Lactobacilli* and *Lachnospiraceae* were significantly decreased. Moreover, the abundance of *Ruminococcaceae* and butyrate-producing bacteria was significantly higher in the *Muc2^−/−^* mice. There were significant differences of gut microbiota between *Muc2^−/−^* and *Muc2^+/+^* mice. The dynamic changes of microbiota might contribute to the development of colitis and colitis-associated colorectal carcinogenesis. Therefore, this study revealed specific functional bacteria in the development of colitis and colitis-associated colorectal carcinogenesis, which will benefit the development of preventive and therapeutic strategies for chronic inflammation and its malignant transformation.

## 1. Introduction

Colorectal cancer (CRC) is the third most common and the second most deadly cancer in the world, and there are about 1.2 million new cases of CRC annually [1]. The lifetime risk of developing CRC in industrialized nations is approximately 5% [2]. Numerous studies have demonstrated that many types of cancers are associated with infections, including viruses, fungi and bacteria, and so on, and about 2 million cancer cases each year are caused by infectious agents [3]. CRC is one of the well-studied cancers and has been known to be closely related with chronic inflammation because of the exposure of intestinal mucosal tissues to enormous numbers of microbes. Recent studies have shown that gut bacteria drive intestinal inflammation and further increase the risk for CRC [4,5,6,7,8].

Accumulating evidence has suggested that the alterations of gut microbiota contribute to the onset of colorectal cancer. For instance, *Escherichia coli* NC101 promote invasive carcinoma in azoxymethane (AOM)-treated *IL-10^−/−^* mice through the polyketide synthase (*pks*) island and colibactin [9], while *Fusobacterium nucleatum* increases proliferation of CRC cells in vitro and tumorigenesis in mice by activating Toll-like Receptor 4 (TLR4) and nuclear factor-κB (NF-κB) signaling pathway and up-regulating expression of microRNA-21 [10]. The microbial dysbiosis, including the increase of some opportunistic pathogens (e.g., *Bacteroides fragilis*, *Enterococcus*, *Escherichia*, *Klebsiella*, *Desulfovibrio*, *Fusobacterium*, *Akkermansia*, etc.) and the decrease of probiotics (e.g., *Lactobacillus*, *Roseburia*, *Ruminococcus*, *Eubacterium*, etc.) have been observed in CRC of animal models and patients [11,12,13,14]. Very recent studies from Flemer et al. have demonstrated that CRC patients can be further stratified based on higher level structures of mucosal-associated bacterial co-abundance groups (CAGs) that resemble the previously formulated concept of enterotypes [15]. Moreover, CRC-associated CAGs were differentially correlated with the expression of host immunoinflammatory response genes [15].

The mucus layer overlying the gut epithelium plays a key role in establishing mutualistic relationship between the host and microbe, and the majority of intestinal mucin is encoded by the *Muc2* gene and secreted by goblet cells. High-sustained levels of tumor necrosis factor-α (TNF-α) and depletion of adherent and goblet cell mucin are necessary for maintenance of acute colitis [16]. Genetic deficiency of the *Muc2* gene results in 90% reduction in mucus and increased exposure of the intestinal epithelial cells to the luminal contents, causing spontaneous colitis and CRC [17,18]. It is well known that intestinal microbiota was crucial for immunological priming, nutrient digestion, mucosal stability, and avoiding pathogenic behavior that might destabilize their host interaction. Commensals that become renegade or a decreased exposure to essential coevolved microorganisms may cause particular health problems such as inflammatory bowel diseases, obesity, or allergies [19]. Therefore, gut microbiome could contribute to the development of CRC in *Muc2^−/−^* mice by regulating metabolic and inflammatory conditions. Morampudi et al. demonstrated that the goblet cell mediator resistin-like molecule-β (RELM-β) drives colitis in *Muc2^−/−^* mice by depleting protective commensal microbes [20]. Using conventionalization of germ-free mice, Huang et al. reported that the absence of *Muc2* converted gut microbiota into a proinflammatory colitogenic phenotype, but pretreatment with dexamethasone to reshape the gut microbiome ameliorated the symptoms of inflammation [21]. However, the detailed changes of gut bacterial community composition in *Muc2^−/−^* mice of different ages are largely unclear.

In this study, we analyzed the changes of gut microbiota in mice of different ages and found that structural segregation of gut microbiota between the *Muc2^−/−^* and *Muc2^+/+^* mice was observed throughout the whole experimental period (from the 48th day to the 178th day). Alpha diversities were higher in the *Muc2^−/−^* mice than those in *Muc2^+/+^* mice. The phylum of Firmicutes was enriched and Bacteroidetes was decreased in *Muc2^−/−^* mice. Interestingly, the abundance of butyrate-producing bacteria was also significantly higher in *Muc2^−/−^* mice than in *Muc2^+/+^* mice. Thus, the changes of gut microbiota in *Muc2^−/−^* mice might contribute to the development of chronic colitis and colorectal tumor formation.

## 2. Results

### 2.1. Histopathology of the Muc2 Mouse Models

As described previously [18,22], *Muc2^−/−^* and *Muc2^+/+^* mice were generated by crossbreeding from *Muc2^+/−^* mice. After weaning (at about 28 days), the mice from each group (i.e., *Muc2^−/−^* and *Muc2^+/+^* mice) at the same age with similar weight (about 18–20 grams) were maintained. Fecal samples were collected at the 48th, 98th, 118th, 138th, and 178th days, and stored at −80 °C. At the age of 178 days, all mice were sacrificed. As reported by us [17,18], all *Muc2^−/−^* mice developed chronic colitis and colorectal hyperplasia and adenomas, and some mice (40%) exhibited colorectal adenocarcinomas at the age of 178 days.

### 2.2. Cytokines Abundances in the Muc2 Mouse Colonic Epithelial Cells

**Figure 1:**
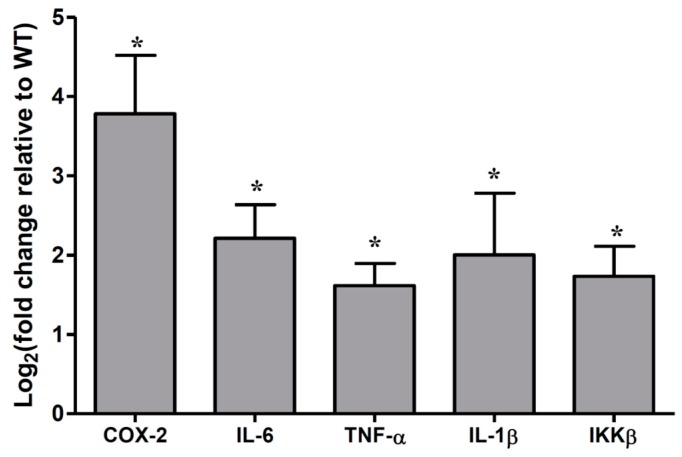
Cytokines were significantly upregulated in colonic epithelial cells of *Muc2^−/−^* mice at the age of 178 days. * *p* < 0.05, compared with *Muc2^+/+^* mice colonic epithelia. The columns were presented as the mean ± SD; *n* = 5 for each group. WT, *Muc2^+/+^* mice. TNF-α—tumor necrosis factor-α.

To determine the association between the microbiota and cytokine changes in colonic epithelial cells, we quantified the frequently changed cytokines in colitis-associated CRC. As shown in Figure 1, the mRNA levels of COX-2, IL-6, TNF-α, IL-1β, and IKKβ were significantly increased in the colonic epithelia cells of *Muc2^−/−^* mice, compared with *Muc2^+/+^* mice; assay by qRT-PCR.

### 2.3. Dynamic Changes of the Diversity Index in Muc2 Mouse Models

**Figure 2:**
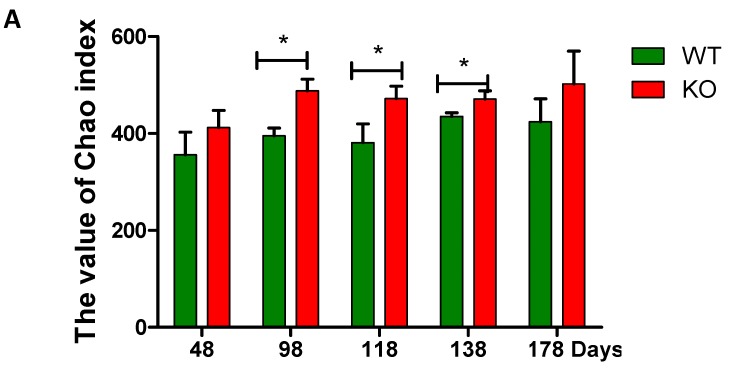

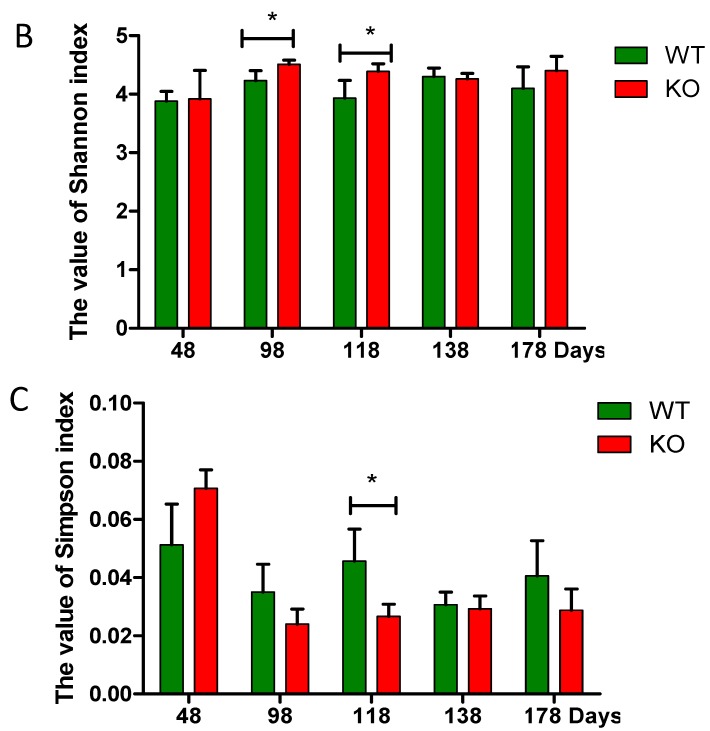
Bacterial diversity analyzed by high-throughput sequencing. (**A**) Chao index; (**B**) Shannon index; (**C**) Simpson index. * indicated significant differences (*p* < 0.05) between *Muc2^−/−^* and *Muc2^+/+^* mice. The results are presented as the mean ± SEM; *n* = 3 for each group. WT, *Muc2^+/+^* mice; KO, *Muc2^−/−^* mice.

Mouse fecal samples were collected at the 48th, 98th, 118th, 138th, and 178th day, and were homogenized bacterial DNA extraction. Then, 16S rRNA was amplified and sequenced. The sequencing results represent the true situation of bacteria because the coverage values were >99% in all the samples. The diversity indexes including Chao, Shannon, and Simpson indices were changed with ages regardless genotypes of the mice (i.e., *Muc2^−/−^* and *Muc2^+/+^* mice) (Figure 2). The Chao and Shannon indices were higher in the *Muc2^−/−^* mice than in the *Muc2^+/+^* mice during the whole experimental period, and significant differences were observed during the experiment (at days 98, 118, and 138 in Chao index, Figure 2A; at days 98 and 118 in Shannon index, Figure 2B). The Simpson index was significantly lower in *Muc2^−/−^* mice at day 118, compared with *Muc2^+/+^* mice (Figure 2C).

### 2.4. Clustering of Bacterial Communities in Muc2^−/−^ Mice

**Figure 3:**
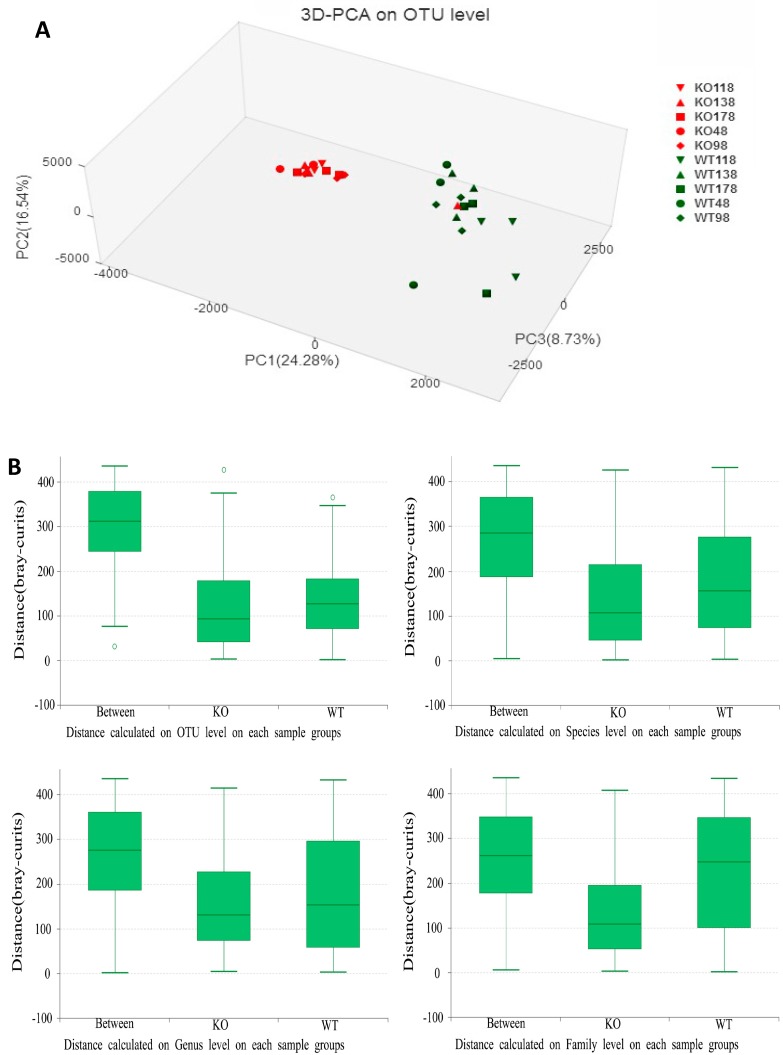
Principal components analysis (PCA) of 16S rRNA gene-sequencing analysis of gut microbes obtained from the *Muc2^−/−^* and *Muc2^+/+^* mice. (**A**) PC1, PC2, and PC3 explain 24.28%, 16.54%, and 8.73% of variation, respectively. WT, *Muc2^+/+^* mice; KO, *Muc2^−/−^* mice. The sample names represented the sampling time and genotypes, e.g., KO48 indicated the samples collected from *Muc2^−/−^* mice at day 48, and WT48 indicated the samples collected from *Muc2^+/+^* mice mouse at day 48. OTU—operational taxonomic units. (**B**) Analysis of similarities (ANOSIM) at different categorical levels were conducted. The middle line in the group “between” were higher than in “KO” (*Muc2^−/−^*) and “WT” (*Muc2^+/+^*) group, so the differences inter-treatment was bigger than that intra-treatment at family, genus, species and OTU levels in *Muc2^−/−^* and ^+/+^ mice. “0” meant the normalization point.

Principal component analysis (PCA) with the taxonomic information at the operational taxonomic unit (OTU) level revealed that the fecal bacterial communities of *Muc2^−/−^* mice were distinct from those of *Muc2^+/+^* mice, except two outliers from *Muc2^−/−^* mouse at days 48 and 138 (Figure 3). The differences were obvious using the principal component scores of principal component 1 (PC1), PC2, and PC3 (24.28%, 16.54%, and 8.73% of represented variances, respectively).

To further test statistically whether there was a significant difference between *Muc2^−/−^* and *Muc2^+/+^* mice, analysis of similarities (ANOSIM) at different categorical levels was conducted (Figure 3B). The *R* values were 0.830, 0.516, 0.460, and 0.355 at the OTU, species, genus, and family level, respectively, and the *p* values were 0.001 at these four categorical levels. The differences inter-treatment were bigger than those intra-treatment at family, genus, species, and OTU levels in *Muc2^−/−^* and *Muc2*^+*/*+^ mice.

**Figure 4:**
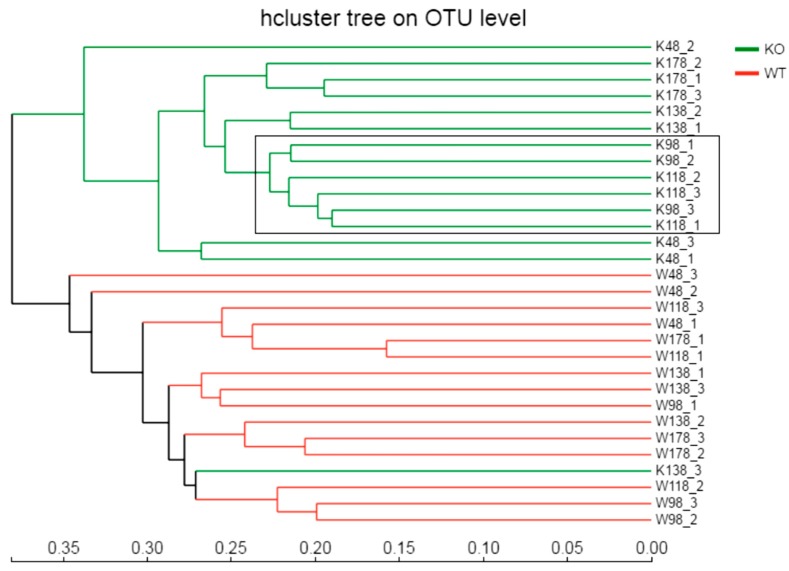
The hierarchical clustering was generated based on the Bray–Curtis method and the difference was significant between *Muc2^−/−^* and *Muc2^+/+^* mice with 16S rRNA gene-sequencing based on OTU levels. WT/W, *Muc2^+/+^* mice; KO/K, *Muc2^−/−^* mice. The sample names at the right side represented the sampling time and genotypes. For example, K48_2 indicated the second sample collected from *Muc2^−/−^* mouse at day 48, and W48_1 indicated the first sample collected from *Muc2^+^*^/*+*^ mice at day 48. Bray–Cutis dendrograms of 16S rRNA gene-sequencing based on OTU level. The number indicated the sampling time, e.g., K48_2 indicated the second sample collected at 48th day from *Muc2^−/−^* mice.

Similarly, the hierarchical clustering was generated based on the Bray–Curtis method and the difference was significant between *Muc2^−/−^* and *Muc2^+^*^/*+*^ mice (Figure 4), except for the third replication of the sample collected on day 138 (K138_3) from *Muc2^−/−^* mice. The PCA and cluster tree indicated that the bacterial communities were separated because of deletion of the *Muc2* gene, and the age did not show the effect on bacterial communities between *Muc2^−/−^* and *Muc2^+/+^* (Figure 4).

### 2.5. Changes of Gut Microbiota at Various Levels in Muc2 Mice

**Figure 5:**
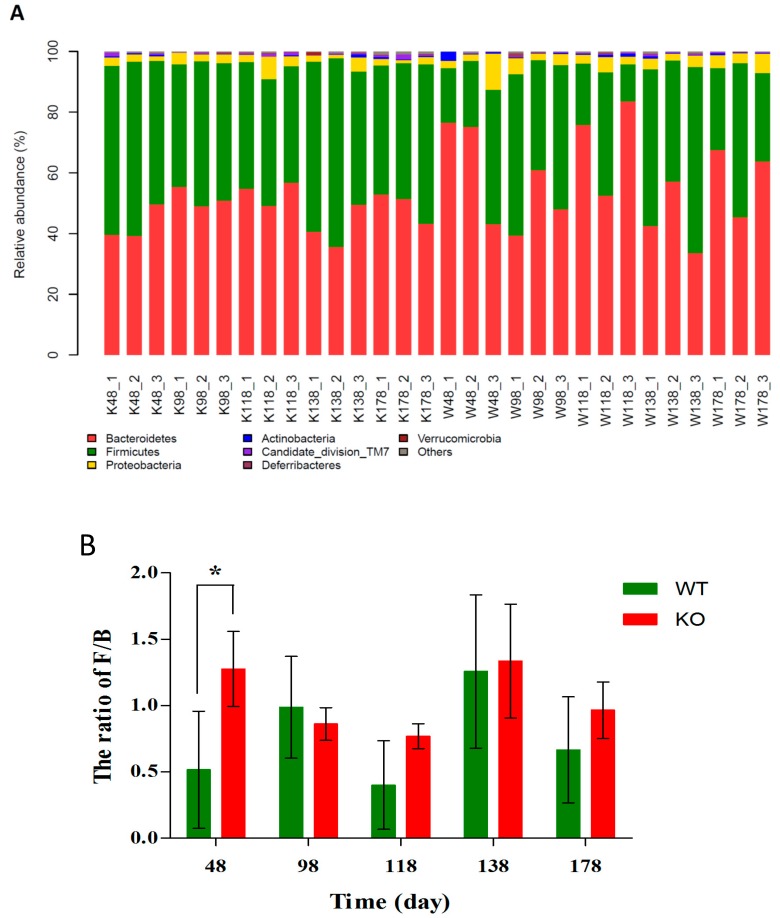
Different community compositions of gut microbes among *Muc2^−/−^* and *Muc2^+/+^* mice at phylum levels. (**A**) Relative abundance of main phyla in the intestinal microbiota; (**B**) The ratio of Firmicutes to Bacteroides (F/B). * indicated the significant differences (*p* < 0.05) between *Muc2^−/−^* and *Muc2^+/+^* mice. W/WT, *Muc2^+/+^* mice; K/KO, *Muc2^−/−^* mice. The sample names represented the sampling time and genotypes, e.g., K48_2 indicated the second sample collected from *Muc2^−/−^* mice at day 48, and W48_1 indicated the first sample collected from *Muc2^+/+^* mice at day 48.

As shown in Figure 5A, the overall bacterial compositions for each sample at the phylum level were presented. Based on the taxonomic results, Bacteroidetes, Firmicutes, and Proteobacteria were the most predominant phyla, the percentages in *Muc2^+/+^* and *Muc2^−/−^* mice were 57.62 ± 15.11 vs. 47.82 ± 6.55, 36.99 ± 14.40 vs. 47.91 ± 7.17, and 4.05 ± 1.49 vs. 2.77 ± 0.95, respectively. The bacterial composition showed high inter-individual variability. Bacteroidetes accounted for 33.54–83.53%, and Firmicutes was 12.25–62.19% among all individual animals. The ratio of Firmicutes to Bacteroidetes (F/B) showed slight differences in *Muc2^−/−^* mouse than that in *Muc2^+/+^* mouse on 98th, 118th, 138th, and 178th day, but the F/B ratio in *Muc2^−/−^* mouse was significantly higher from that in the *Muc2^+/+^* mice at day 48 (Figure 5B).

**Figure 6:**
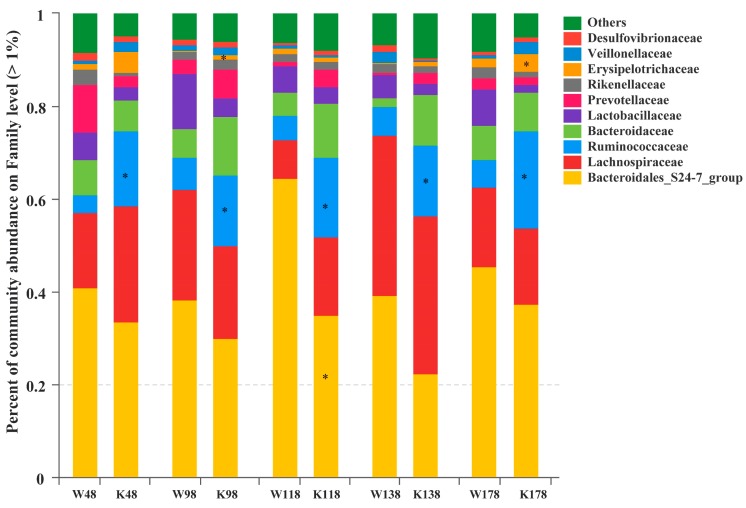
Relative abundance of the families exceeding 1% in *Muc2^−/−^* and *Muc2^+/+^* mice at different time points. * indicated the significant differences (*p* < 0.05) between *Muc2^−/−^* and *Muc2^+/+^* mice. W, *Muc2^+/+^* mice; K, *Muc2^−/−^* mice. The sample names at the x axis represented the sampling time and genotypes. For example, W48 indicated the samples collected from *Muc2^+/+^* mice at day 48, and K48 indicated the samples collected from *Muc2^−/−^* mice at day 48.

At the family level, Bacteroidales_S24-7_group, *Lachnospiraceae*, *Ruminococcaceae*, *Bacteroidaceae*, *Lactobacillaceae*, *Prevotellaceae*, *Rikenellaceae*, *Erysipelotrichaceae*, *Veillonellaceae*, and *Desulfovibrionaceae* were the most abundant families that exceeded 1% (Figure 6). It was interesting that the Ruminococcaceae family was always enriched significantly in *Muc2^−/−^* mice compared with that in *Muc2^+/+^* mice, and the Erysipelotrichaceae family was more abundant at days 98 and 178. The relative abundance of Bacteroidales_S24-7_group was much lower in *Muc2^−/−^* mice at day 118, compared with the *Muc2^+/+^* mice.

**Figure 7:**
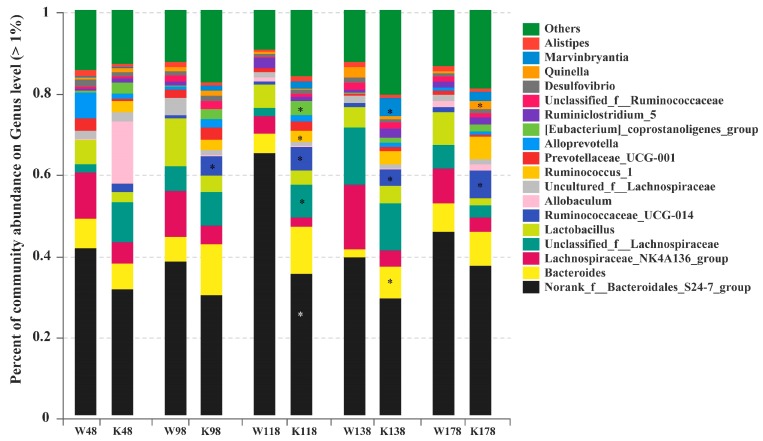
Relative abundance of genus exceeding 1% in the *Muc2^−/−^* and *Muc2^+/+^* mice at genus level and at different time points. * indicated the significant differences (*p* < 0.05) between *Muc2^−/−^* and *Muc2^+/+^* mice. W, *Muc2^+/+^* mice; K, *Muc2^−/−^* mice. The sample names at the *x* axis represented the sampling time and genotypes. For example, W48 indicated the samples collected from *Muc2^+/+^* mice at day 48, and the K48 indicated the samples collected from *Muc2^−^*^/*−*^ mice at day 48.

As shown in Figure 7, there were a total of 18 genera that exceeded 1% of the total bacteria at the genus level, and the most abundant genera were norank_f_Bacteroidales_S24-7_group, *Bacteroides*, Lachnospiraceae_NK4A136_group, unclassified_f_Lachnospiraceae, and *Lactobacillus*. In those predominant 18 genera, no significant differences were observed at day 48 between *Muc2^−/−^* and *Muc2^+/+^* mice. However, compared with the *Muc2^+/+^* mice, Ruminococcaceae_UCG-014 was enriched significantly at days 98, 118, 138, and 178 (0.61% vs. 4.75% at day 98, 0.51% vs. 6.37% at day 118, 0.35% vs. 3.81% at day 138, 0.83% vs. 7.56% at day 178). Unclassified_f_Lachnospiraceae (2.20% vs. 8.51%), Ruminococcus_1 (0.20% vs. 3.64%), and [Eubacterium] coprostanoligenes_group (0.01% vs. 2.98%) were significantly enriched at day 118. *Bacteroides* and *Marvinbryantia* were more abundant at day 138 (2.09% vs. 8.37%, 0.27% vs. 4.58%, respectively), and *Quinella* was enriched at day 178 (0.58% vs. 2.01%). The relative abundance of nonrank_f_Bacteroidales_S24-7_group was significantly lower at the 118th day in the *Muc2^−/−^* mice than that in the *Muc2^+/+^* mice (62.85% vs. 35.30%). All the significantly changed genera between *Muc2^−/−^* and *Muc2^+/+^* mice were listed in Supplementary Table S1, in which, 6, 27, 24, 11, and 24 significantly changed genera were detected at days 48, 98, 118, 138, and 178, respectively.

**Figure 8:**
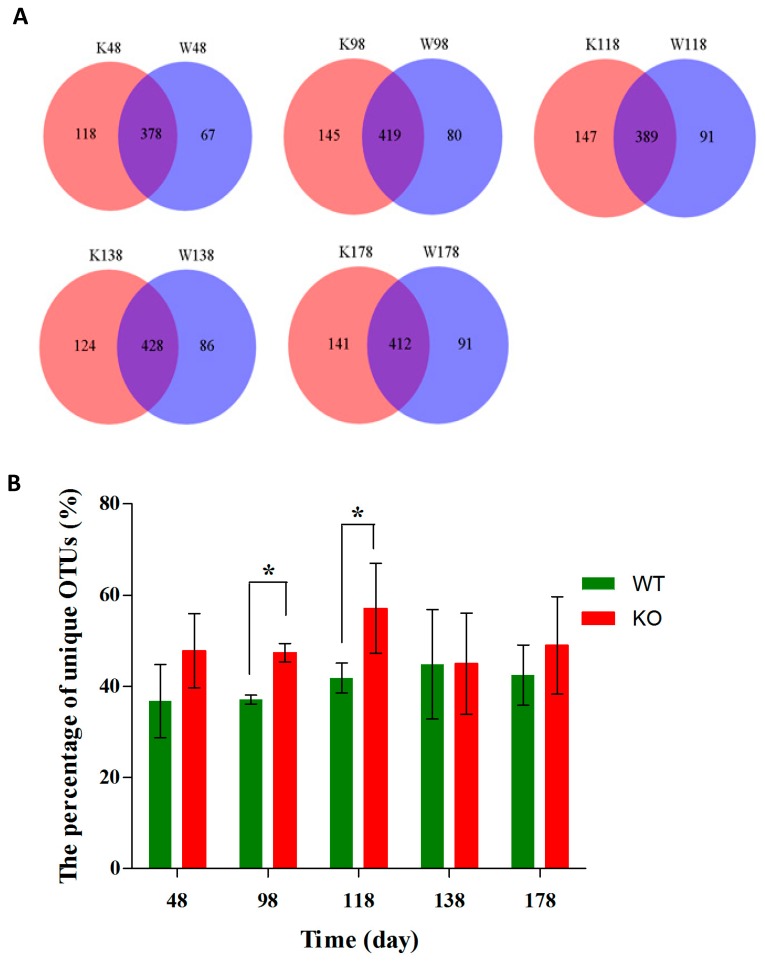
Different community structures of gut microbes among *Muc2^−/−^* and *Muc2^+/+^* mice at OTU level and at different time points. (**A**) The Venn diagrams showed the common and unique OTUs at different time points; (**B**) the percentage of unique OTUs. * indicated the significant differences (*p* < 0.05) between *Muc2^−/−^* and *Muc2^+/+^* mice. W, *Muc2^+/+^* mice; K, *Muc2^−/−^* mice. The sample names represented the sampling time and genotypes. For example, W48 indicated the samples collected from *Muc2^+/+^* mice at day 48, and the K48 indicated the samples collected from *Muc2^−/−^* mice at day 48.

At the OTU level, as shown in Figure 8A, there were more unique OTUs in the *Muc2^−/−^* mice than those in *Muc2^+/+^* mice at day 48 (118 vs. 67), day 98 (145 vs. 80), day 118 (147 vs. 91), day 138 (124 vs. 86), and day 178 (141 vs. 91). Moreover, at days 98 and 118, the percentage of unique OTUs were significantly larger in *Muc2^−/−^* mice than those in *Muc2^+/+^* mice (Figure 8B).

**Figure 9:**
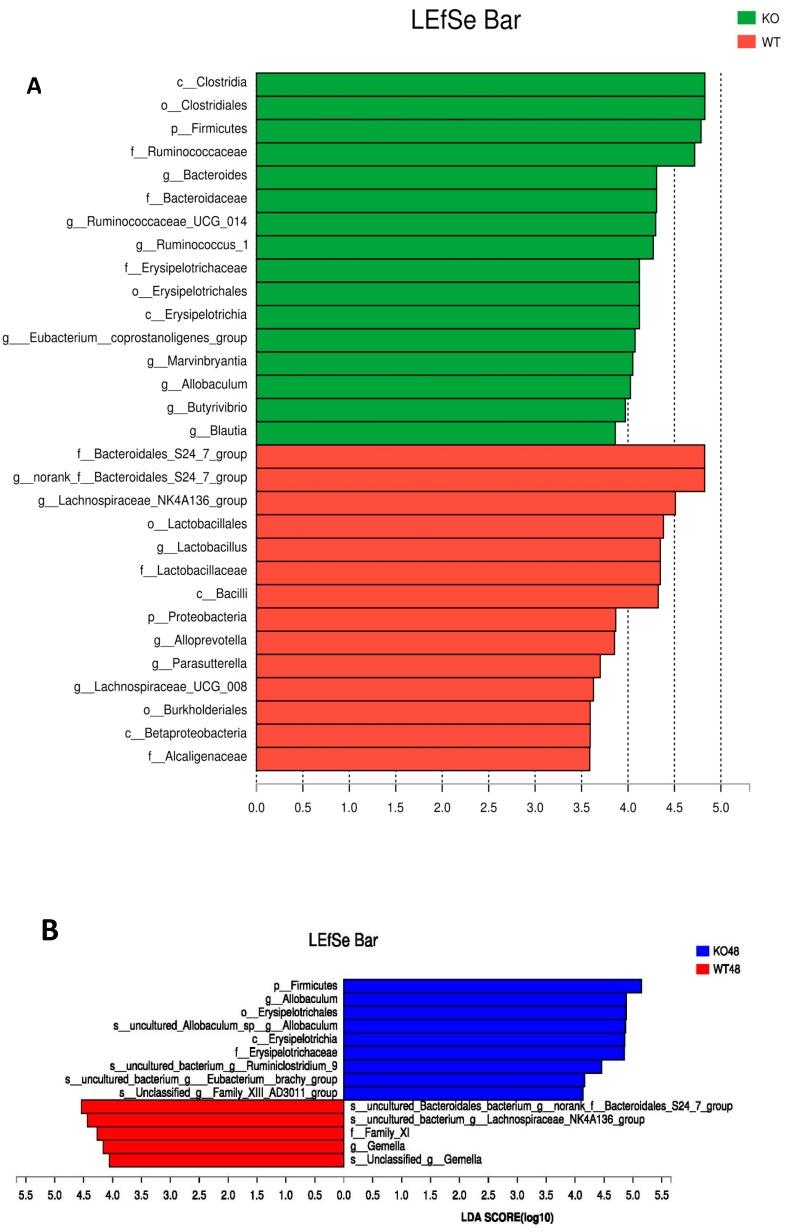

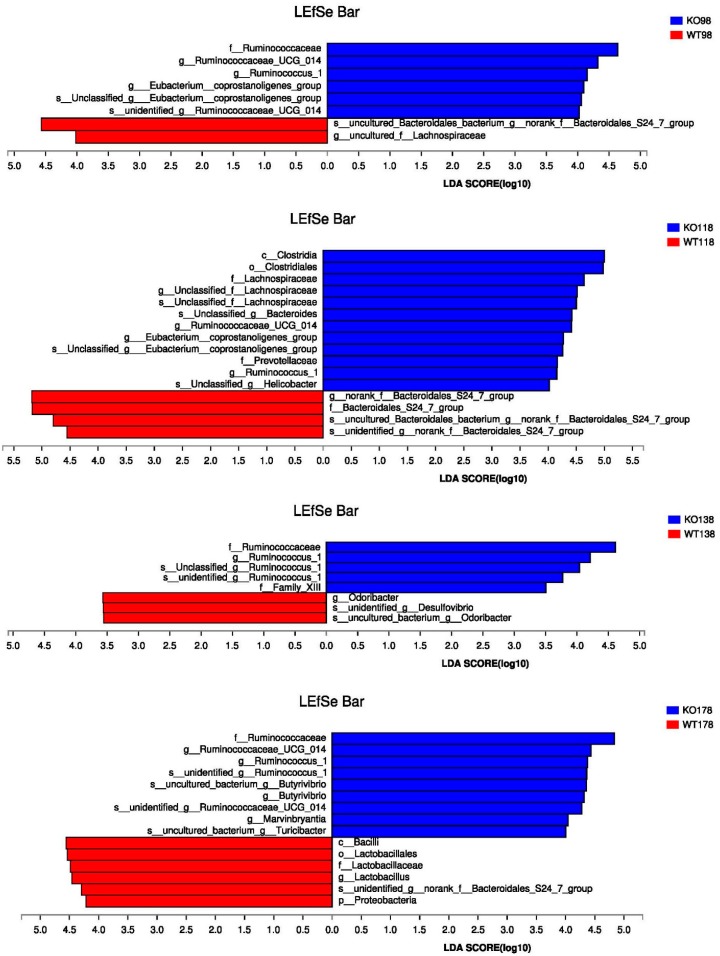
Linear discriminant analysis (LDA) coupled with effect size measurement (LEfSe) analysis of gut microbes among *Muc2^−/−^* and *Muc2^+/+^* mice. (**A**) Histogram of the linear discriminant analysis (LDA) scores for differentially abundant genera among *Muc2^−/−^* and *Muc2^+/+^* mice; (**B**) the LEfSe analysis at different time points. WT, *Muc2^+/+^* mice; KO, *Muc2^−/−^* mice.

LaLEfSe analysis was then performed to obtain the cladogram representation and the characteristic bacteria of the gut microbiota within the *Muc2^−/−^* and *Muc2^+/+^* mice. As shown in Figure 9A, the greatest differences in taxa between the two communities were detected by linear discriminant analysis (LDA). In *Muc2^−/−^* mice, the *Clostridiales*, *Ruminococcaceae*, *Bacteroides*, *Erysipelotrichaceae*, Eubacterium_ coprostanoligenes_group, *Marvinbryantia*, *Allobaculum*, *Butyrivibrio*, and *Blautia* were the characteristic bacteria; whereas in *Muc2^+/+^* mice, f_Bacteroidales_S24_7_group, Lachnospiraceae_NK4A136_group, *Lactobacillales*, *Lactobacillus*, *Lactobacillaceae*, *Alloprevotella*, *Parasutterella*, *Lachnospiraceae_UCG_008*, and *Alcaligenaceae* were more characteristic. All of them were key phylotypes involved in the segregation of gut microbiota in *Muc2^−/−^* and *Muc2^+/+^* mice in accordance with the linear discriminant analysis (LDA) coupled with effect size measurement (LEfSe) analysis.

The LEfSe analysis was also conducted at different time points (Figure 9B). At the 48th, 98th, 138th, and 178th days, Ruminococcaceae was the most significantly characteristic bacteria in the *Muc2^−/−^* mice, while f_Bacteroidales_S24_7_group was the significantly characteristic bacteria in the *Muc2^+/+^* mice. The characteristic bacteria changed with the age. In the *Muc2^−/−^* mice, *Erysipelotrichaceae* was characteristic at the 48th day, whereas [Eubacterium]_coprostanoligenes_group was characteristic at the 98th and 118th day, and *Marvinbryantia*, *Butyrivibrio*, and *Turibacter* on 178th day. In the *Muc2^+^*^/*+*^ mice, *Lactobacillus* was characteristic at the 178th day.

Interestingly, *Roseburia*, *Butyricimonas*, *Coprococcus*, *Clostridium*, *Ruminococcus*, and *Eubacterium* detected in this study are, in fact, butyrate-producing bacteria in the gut. As shown in Table 1, the total abundances of butyrate-producing bacteria were significantly higher in *Muc2^−/−^* mice than those in *Muc2^+/+^* mice at the days 48, 98, 118, 138, and 178. (7.50% vs. 1.45%, 10.65% vs. 0.77%, 7.58% vs. 0.57%, 4.85% vs. 2.93%, 8.04% vs. 0.55%, respectively). Moreover, *Clostridium* and *Butyricimonas* were detected in the *Muc2^−/−^* mice all the time, no *Clostridium* was detected in *Muc2^+/+^* mice, and *Butyricimonas* was observed at days 48 and 98 in the *Muc2^+/+^* mice.

Lastly, the relative abundance of some bacteria was obtained after stratification and analysis. As shown in Table 2, *Desulfovibrio* and *Turicibacter* were always significantly higher in *Muc2^−/−^* mice than those in *Muc2^+/+^* mouse, and *Akkermansia* and *Escherichia* were significantly enriched in *Muc2^−/−^* mice at the days 98, 118, 138, and 178. No significant differences of *Enterococcus* were observed between *Muc2^−/−^* and *Muc2^+/+^* mice.

## 3. Discussion

Intestinal dysbiosis or the changes of gut microbe has been reported to play a crucial role in the development of colitis and malignant transformation to CRC [7], but the dynamic changes of gut microbiota during lesions progression are unclear. Our previous studies have reported that genetic deletion of *Muc2* gene spontaneously causes colitis before three months of age and then progresses to CRC [17,18], which was linked to the activation of inflammatory signaling and epigenetic alterations, such as differential expression of microRNAs. Herein again, we found that changes of gut microbiota were also associated with the alterations of cytokines. The present study provided direct evidence that the alterations of intestinal microbiota correlated with colonic lesions and progression. Through community structural and LEfSe analysis, we have found that loss of the *Muc2* gene could induce significant segregation of gut microbiota in *Muc2^−/−^* mice from *Muc2^+/+^* mice at the age of 48 days, much earlier than colorectal carcinogenesis occurs. These findings strongly suggested that gut bacterial composition in *Muc2^−/−^* mice was not only altered by pathophysiological changes, but also involved in the development of diseases, which is consistent with the conclusion of previous studies. Klimesova et al. (2013) found that antibiotic treatment reduced the incidence and severity of tumors in AOM/dextran sodium sulfate (DSS) induced CAC mice [28]. Colitis also can promote tumorigenesis by altering microbial composition and inducing the expansion of microorganisms with genotoxic capabilities [9]. Core 1- and core 3-derived mucin-type O-linked oligosaccharides (*O*-glycans) are major components of the colonic mucus layer. The study of Bergstrom et al. (2016) also indicated that intestinal bacteria involved in the mucin depletion induced colitis and colorectal cancer, in which antibiotic depletion of the microbiota reduced the development of colitis and cancer formation in mice that lack core 1- and core 3-derived intestinal *O*-glycans [29].

It is thought that high diversity of bacteria is beneficial to the gut ecosystem. However, in this study, we found that the Chao and Shannon diversity indices were higher in *Muc2^−/−^* mice than in *Muc2^+/+^* mice, in terms of increase of unique OTUs, decrease of some of predominant genus and enrichment of minorities, and finally the increase of diversity, in *Muc2^−/−^* mice. It is notable that some of enriched genus in the *Muc2^−/−^* mice were potential pathobionts, such as *Turicibacter*, *Akkermansia*, and *Desulfovibrio*, which was consistent with the report of previous studies that the alpha diversity of gut microbiome was higher in CRC patients than in healthy subjects [14,30,31]. The significantly higher diversity of the gut microbiota was also observed in Parkinson’s disease patients compared with that of the healthy group [32].

We also found that the phylum of Firmicutes was enriched and the Bacteroidetes was decreased in the *Muc2^−/−^* mice, consistent with previous studies on CRC mouse models and patients [13,27], resulting largely from the increase of Ruminococcaceae and Erysipelotrichaceae, which belonged to Firmicutes, and from the decrease of Bacteroidales_S24-7_group, which belonged to Bacteroidetes at family level in the *Muc2^−/−^* mice. The most predominant genera enriched in the *Muc2^−/−^* mice also belong to Firmicutes, including Ruminococcaceae_UCG-014, unclassified_f_Lachnospiraceae, Ruminococcus_1, [Eubacterium] coprostanoligenes_group, *Marvinbryantia*, and *Quinella*.

Bacterial composition imbalance analysis showed that multiple potential pathobionts were increased and probiotic species were decreased within the microbiota of *Muc2^−/−^* mice. For example, *Desulfovibrio* has been reported to cause DNA damage and genomic instability and the cumulative mutations observed in CRC through the production of hydrogen sulfide by reducing sulfate [12,14,33], and pathogenic *Escherichia coli* promotes colon carcinogenesis through the *pks* island and colibactin [9]. Gagniere et al. have also reported that colonization of pathogenic *Escherichia coli* in colonic mucosa could be involved in the development of CRC, differences in the involvement of colibactin-producing *Escherichia coli* in colorectal carcinogenesis according to the CRC phenotype [26]. *Akkermansia* was positively correlated with colonic tumor multiplicity and size and was significantly increased in CRC mouse and patients, although *Akkermansia municiphila* is a member of healthy gut microbiome and potential probiotic [23,24,27,34]. *Turicibacter* was another potential bacteria and has been reported to increase the carcinogen AOM/DSS-induced colitis-associated CRC [27]. In this study, the abundance of *Desulfovibrio*, *Escherichia*, *Akkermansia*, and *Turicibacter* was significantly higher in *Muc2^−/−^* mice than in *Muc2^+/+^* mice at the 98th, 118th, 138th, and 178th day. Previous studies have demonstrated that *Enterococcus faecalis* could cause the colorectal carcinogenesis through double-strand DNA breaks and chromosome instability, and the enrichment of *Enterococcus* was observed in CRC patients [25,35,36]. However, no significant difference of *Enterococcus* abundance was seen between *Muc2^−/−^* and *Muc2^+/+^* mice in this study.

The LEfSe analysis showed that *Clostridiales* and *Lactobacillaceae* were the predominant bacteria in *Muc2^−/−^* and *Muc2^+/+^* mice, respectively, which was consistent with the previous study [21]. Morampudi et al. also reported a selective loss of colonic *Lactobacilli* spp. in *Muc2^−/−^* mice, and that oral replenishment with murine *Lactobacillus* spp. ameliorated their spontaneous colitis [20]. Chen et al. found that NLRP12 (which encodes a negative regulator of innate immunity) deficiency in mice led to increased colonic inflammation, causing a loss of protective gut commensal strains (of the family Lachnospiraceae) and a greater abundance of colitogenic strains (of the family Erysipelotrichaceae) [37]. The LEfSe analysis in this study also revealed the predominance of Erysipelotrichaceae in *Muc2^−/−^* mice, and *Lactobacilli* and Lachnospiraceae in *Muc2^+/+^* mice.

Ruminococcaceae is an important family that is always predominant in the *Muc2^−/−^* mice. Berry et al. reported that Ruminococcaceae was increased in relative abundance in dextran sodium sulfate (DSS)-induced colitis mice [38], and Sun et al. also reported an increased trend of Ruminococcaceae during the CRC process induced by 1,2-dimethylhydrazine [39]. However, recent studies have shown that Ruminococcaceae enriched in the mice were less sensitive to colitis and control healthy human subjects compared with gut disease, including Crohn’s disease, ulcerative colitis, and pseudomembranous colitis [40,41]. Thus, the roles of Ruminococcaceae in colitis and CRC are uncertain and need to be further investigation.

Butyrate, a four-carbon fatty acid, is formed in the human colon by bacterial fermentation of carbohydrates including dietary fiber. Numerous studies have suggested that butyrate-producing bacteria is beneficial to intestinal health, and the bacteria was reduced in CRC patients [23,42] and in mice with CRC induced by AOM/DSS or 1,2-dimethylhydrazine (1,2-DMH) [12,27]. However, not all studies support the chemo-preventive effect of butyrate, and the lack of agreement in in vivo and in vitro studies on butyrate and colon cancer has been termed the “butyrate paradox” [43,44,45,46]. Belcheva et al. reported that gut microbes induced CRC by providing butyrate that fuel hyperproliferation and transformation of *Msh2^−/−^* colon epithelial cells, but the effects of butyrate largely depend on the concentrations and models [45,47]. In this study, the relative abundance of butyrate-producing bacteria was always higher in *Muc2^−/−^* mice than in *Muc2^+/+^* mice, suggesting that the role of butyrate and butyrate-producing bacteria in gut health should be carefully considered. The content of butyrate in gut should be directly detected in the further study.

Several rodent models of CRC have been developed, such as genetic deficient models (e.g., *Muc2^−/−^*, *Msh2^−/−^*, *IL10^−/−^*, *p53^−/−^*, etc.), chemical carcinogen-induced models (e.g., AOM/DSS, 1,2-DMH), and inoculation models [48], to evaluate features of CRC in humans, and have been widely used to determine the underlying mechanisms of carcinogenesis and to evaluate the effective prevention and therapy, but none of them is a perfect model. However, the *Muc2^−/−^* mouse model could spontaneously develop chronic colitis and CRC, and the histopathological change is highly similar to the development process of colitis-associated CRC of human; particularly, the histopathology of colonic inflammation is also similar to human ulcerative colitis [17,18]. The gut microbiota changes of *Muc2^−/−^* mouse in this study were also consistent with the bacterial changes observed in CRC patients [13,14,31], such as the higher alpha diversity, the increase of Firmicutes and decrease of Bacteroidetes, the enrichment of potential pathobionts (e.g., *Desulfovibrio*, *Escherichia*, and Erysipelotrichaceae), and the reduction of probiotics (e.g., *Lactobacilli*) and *Lachnospiraceae*. Therefore, the *Muc2^−/−^* model is a useful model to study human rectal cancer, especially for colitis-associated CRC.

In conclusion, there were significant differences of gut microbiota between *Muc2^−/−^* and *Muc2^+/+^* mice, and the dynamic changes of microbiota might contribute to the development of colitis and colitis-associated colorectal carcinogenesis. Therefore, this study revealed the dynamic changes of specific functional bacteria in the development of colitis and colitis-associated colorectal carcinogenesis, which is benefit to the development of preventive and therapeutic strategies for chronic inflammation and its malignant transformation.

## 4. Materials and Methods

### 4.1. Mouse Models, Fecal Samples Collection, and Histopathology

As described previously [18,22], *Muc2^−/−^* and *Muc2^+/+^* mice were generated by crossbreeding from *Muc2^+/−^* mice. Genotypes of the offspring were determined using PCR and *Muc2* specific gene primers using the DNA extracted from mouse tails at the age of 10 days old. After weaning (at about 28 days), five male mice from each group (i.e., *Muc2^−/−^* and *Muc2^+/+^* mice) at the same age with similar weight (about 18–20 grams) were maintained, and housed separately according to genotype. All mice were raised in sterilized cages under controlled conditions (i.e., temperature 23 ± 2 °C, humidity 55 ± 5%, and 12 h light/dark cycles), and fed with standard rodent chow food and sterilized water under specific pathogen-free conditions. Fecal samples were collected at the 48th, 98th, 118th, 138th, and 178th days, and stored at −80 °C. At the age of 178 days, all mice were sacrificed, the entire colons were dissociated and washed with cold PBS, tumorigenesis was examined under dissecting microscopy, and about 1 cm section of colon was cut and immediately put in 10% buffered formalin for fixation. The remaining colon tissue was used to isolate epithelial cells, as reported by us [17]. The isolated colonic epithelial cells were stored at −80 °C for further study. The fixed colon tissues were embedded in paraffin, sectioned, and stained with hematoxylin and eosin for microscopic examination.

### 4.2. The Analysis of Cytokines in Colonic Epithelial Cells by Quantitative RT-PCR

The total mRNA of colonic epithelial cells was extracted using Trizol reagent (Takara, Dalian, China), as per manufacturer’s instruction, and reverse transcription was conducted using Reverse Transcription Kit (Takara, Dalian, China). The quantity and quality of cDNA was evaluated by 1% (*w*/*v*) agarose gel electrophoresis in 0.5 mg/mL ethidium bromide and Nano Drop 2000 ultraviolet spectrophotometry (Thermo Fisher Scientific, Wilmington, DE, USA). Real-time quantitative PCR (qPCR) was performed using the StepOne System (ABI), and the fold changes of expression levels was calculated as reported by us [17].

### 4.3. High-throughput Sequencing and Bioinformatics Analysis

Six fecal samples (i.e., three from *Muc2^−/−^* and three from *Muc2^+/+^* mice) were randomly collected, and were homogenized with a bead-based technique on a FastPrep-24. Total bacterial DNA from the fecal samples were extracted using a FastPrep-24 system (MP Biomedicals, Santa Ana, CA, USA) and QIAamp DNA Stool Mini Kit (Qiagen, Hilden, Germany), and evaluated by 1% (*w*/*v*) agarose gel electrophoresis. PCR was performed with the following universal 16S rRNA primers (V3–V5 region): 338F (5′-ACTCCTACGGGAGGCAGC-3′) and 806R (5′-GGACTACHVGGGTWTCTAAT-3′) [49]. Both primers were linked to an Illumina sequencing adapter, and the reverse primer contained a sample barcode. PCR products were purified and the concentrations were adjusted for sequencing on an Illumina Miseq PE300 system (MajorBio Co., Ltd., Shanghai, China). The raw data were deposited in Sequence Read Archive (SRA). The access number is SRP123270 (https://www.ncbi.nlm.nih.gov/sra/?term=srp123270).

Quality control of DNA was performed by fragment analyzer on the pooled amplicon library to confirm correct amplicon size. FLASH software was used for assemblage of contiguous sequences and removal if found to be short after trimming for a base quality below 30. The uparse method (http://www.drive5.com/uparse/) was used to cluster contigs and remove chimera using version 7 of usearch (http://www.drive5.com/usearch/). The optimized sequences were clustered into operational taxonomic units (OTUs) with 97% similarity and aligned using SILVA database (http://www.arbsilva.de/). In total, 1,126,145 valid and trimmed sequences were obtained from all 30 samples, with an average length of 439 bp per sequence. The total numbers of OTUs at 97% similarity level were 713. The minimum number of reads (30752 reads) sub-sample was taken from each sample for subsequent analysis. The data were analyzed on the free online platform of Majorbio I-Sanger Cloud Platform (www.i-sanger.com). The alpha diversity analysis was performed using Mothur software package (http://www.mothur.org/wiki/Main_Page). Bray–Curtis similarities were used to construct a cluster dendrogram. Unweighted Unifrac distance metrics analysis was conducted using OTUs from each sample, and the principal component analysis in terms of the matrix of distance was performed. A metagenomic biomarker discovery approach was employed with LEfSe (linear discriminant analysis (LDA) coupled with effect size measurement), which performed a nonparametric Wilcoxon sum-rank test followed by LDA analysis using online software (http://huttenhower.sph.harvard.edu/galaxy/) to assess the effect size of each differentially abundant taxon [50].

### 4.4. Statistical Analysis

The results were expressed as means ± SD (i.e., standard deviation) in individual experiments. SPSS statistical software (SPSS 19.0, Chicago, IL, USA) was used to perform data analysis. Differences in the diversity index, richness, and cytokine abundance were calculated by Student’s *t*-test, χ^2^-test, or analysis of variances where appropriate. *p* < 0.05 was considered statistically significant with two-side statistical analysis.

### 4.5. Ethics Statement

This study was carried out in accordance with the recommendations of the Institute Animal Care and Use Committee of Xinxiang Medical University, China. The experimental protocol for animal studies was reviewed and approved by Institute Animal Care and Use Committee of Xinxiang Medical University, China. (Protocol #20121226-02 was approved on December 26, 2012).

## Figures and Tables

**Table 1:** Relative abundance of butyrate-producing bacteria.

Relative Abundance (%)		Genus						
		*Roseburia* (×10^−2^)	*Butyricimonas* (×10^−2^)	*Coprococcus* (×10^−2^)	*Clostridium* (×10^−2^)	*Ruminococcus*	*Eubacterium*	Total
D48	WT	5.5 ± 1.12	1.9 ± 0.35	0.5 ± 0.14	0	0.83 ± 0.13	0.54 ± 0.27	1.45 ± 0.031
	KO	85.3 ± 19.80	5.3 ± 1.33	8.8 ± 0.97	30.3 ± 0.22	3.23 ± 0.57	2.97 ± 1.17	7.50 ± 1.39 *
D98	WT	50.4 ± 9.79	0.5 ± 0.08	6.7 ± 0.91	0	0.090 ± 0.031	0.14 ± 0.092	0.77 ± 0.15
	KO	216.8 ± 3.72	9.8 ± 2.22	43 ± 3.59	52.8 ± 1.51	3.30 ± 0.98	4.12 ± 1.34	10.65 ± 1.36 *
D118	WT	38.1 ± 7.33	0	4.1 ± 0.72	0	0.01 ± 0.008	0.14 ± 0.014	0.57 ± 0.14
	KO	58.3 ± 12.08	7.9 ± 1.71	13 ± 3.34	3.8 ± 0.79	3.06 ± 0.82	3.70 ± 0.67	7.58 ± 1.28 *
D138	WT	202.9 ± 30.85	0	6.7 ± 1.47	0	0.02 ± 0.0056	0.08 ± 0.04	2.93 ± 0.44
	KO	24.2 ± 3.91	4.5 ± 0.88	12 ± 2.58	1.9 ± 0.19	3.30 ± 0.74	1.12 ± 0.47	4.85 ± 0.97 *
D178	WT	33.3 ± 2.82	0	4.0 ± 0.81	0	0.02 ± 0.0069	0.17 ± 0.07	0.55 ± 0.058
	KO	51.2 ± 11.40	4.1 ± 0.76	7.2 ± 0.2	0.9 ± 0.00	5.89 ± 0.93	1.52 ± 0.81	8.04 ± 1.34 *

Note: D48 indicated that the samples were collected at Day 48, and so on. WT, *Muc2^+/+^*, KO, *Muc2^−/−^.* * *p* < 0.05, comparison between *Muc2^−/−^* and *Muc2^+/+^* mice.

**Table 2:** Relative abundance of potential pathogens.

Relative Abundance (%)		Genus				
		*Akkermansia* (×10^−3^) [23,24]	*Desulfovibrio* (×10^−3^) [12,14]	*Enterococcus* (×10^−24^) [13,25]	*Escherichia* (×10^−4^) [9,14,26]	*Turicibacter* (×10^−2^) [27]
D48	WT	0	0.47 ± 0.35	4.7 ± 1.42	100.1 ± 61.3 *	0.38 ± 0.06
	KO	0	8.0 ± 1.91 *	3.5 ± 0.27	0	12.4 ± 5.21 *
D98	WT	0	0.12 ± 0.09	1.2 ± 0.89	0.3 ± 0.18	0
	KO	0.95 ± 0.37 *	13.2 ± 5.7 *	5.2 ± 3.59	15.4 ± 0.011 *	5.7 ± 0.98 *
D118	WT	0	7.1 ± 3.28	5.2 ± 3.66	1.4 ± 0.98	0
	KO	5.2 ± 0.13 *	14.8 ± 2.1 *	1.7 ± 1.26	18.0 ± 7.88 *	7.8 ± 5.26 *
D138	WT	0	8.4 ± 3.60	6.9 ± 3.47	2.9 ± 1.47	0
	KO	3.5 ± 0.39 *	14 ± 2.47 *	1.8 ± 1.55	3.4 ± 2.51	0.7 ± 0.28 *
D178	WT	0	6.7 ± 2.06	1.9 ± 0.81	6.6 ± 3.58	0
	KO	0.95 ± 0.11 *	11.3 ± 0.81 *	1.5 ± 0.19	1.7 ± 1.30	2.2 ± 0.92 *

Note: D48 indicated that the samples were collected at Day 48, and so on. WT, *Muc2^+/+^,* KO, *Muc2^−/−^* mice. The number in [] stood for the related references. * *p* < 0.05, comparison between *Muc2^−/−^* and *Muc2^+/+^* mice.

## Supplementary Materials

Supplementary materials can be found at http://www.mdpi.com/1422-0067/19/9/2809/s1.
