# Supplementary material for: The Dynamic Changes of Gut Microbiota in Muc2 Deficient Mice
Source: Int J Mol Sci. 2018 Sep 18;19(9):2809. doi: 10.3390/ijms19092809 (PMC6164417; doi:10.3390/ijms19092809)
Supplement: Supplementary file 1 [file ijms-19-02809-s001.pdf]

Supplementary Table 1. Genera that differed significantly between *Muc2*$^{-/-}$ and $^{+/+}$ mice

Time	Phylum	Genus	Relative abundance (%)		*p*-value	Directions of change
			Muc2$^{+/+}$	*Muc2*$^{-/-}$		
Day 48	Bacteroidetes	*Odoribacter*	1.24 ± 0.57	0.041 ± 0.014	0.02175	↓
	Firmicutes	*Turicibacter*	0.0038 ± 0.00065	0.12 ± 0.052	0.01769	↑
	Firmicutes	*[Eubacterium]_brachy_group*	0.011 ± 0.0076	0.045 ± 0.0086	0.007038	↑
	Firmicutes	*Family_XIII_AD3011_group*	0.0012 ± 0.0021	0.046 ± 0.026	0.04113	↑
	Proteobacteria	*Desulfovibrio*	0.00047 ± 0.00035	0.0080 ± 0.0019	0.03126	↑
	Proteobacteria	*Escherichia*	0.010 ± 0.0061	0	0.001869	↓
Day 98	Firmicutes	*Ruminococcaceae_UCG-014*	0.6141 ± 0.42	4.75 ± 0.86	0.001718	↓
	Firmicutes	*uncultured_f__Ruminococcaceae*	1.38 ± 0.20	0.59 ± 0.13	0.004471	↓
	Proteobacteria	*Parasutterella*	0.57 ± 0.15	0.060 ± 0.029	0.004093	↓
	Firmicutes	*Ruminococcaceae_UCG-013*	0.40 ± 0.16	0.080 ± 0.048	0.03257	↓
	Firmicutes	*norank_f__Clostridiales_vadinBB60_group*	0.28 ± 0.12	0.045 ± 0.028	0.02807	↓
	Firmicutes	*Lachnospiraceae_UCG-008*	0.0089 ± 0.0043	0	0.02312	↓
	Firmicutes	*Butyrivibrio*	0.0013 ± 0.00026	1.23 ± 0.46	0.01017	↑
	Firmicutes	*Blautia*	0.015 ± 0.011	1.02 ± 0.55	0.03343	↑
	Firmicutes	*Lachnoclostridium*	0.31 ± 0.022	0.63 ± 0.17	0.03047	↑
	Firmicutes	*Romboutsia*	0.0063 ± 0.0043	0.87 ± 0.46	0.03042	↑
	Bacteroidetes	*Parabacteroides*	0.15 ± 0.096	0.50 ± 0.16	0.0319	↑
	Firmicutes	*Lachnospiraceae_UCG-010*	0	0.65 ± 0.18	0.003141	↑
	Firmicutes	*Clostridium_sensu_stricto_1*	0	0.50 ± 0.28	0.03646	↑
	Proteobacteria	*Sutterella*	0.094 ± 0.067	0.38 ± 0.15	0.03902	↑
	Firmicutes	*Family_XIII_AD3011_group*	0.0064 ±0.0058	0.21 ± 0.076	0.01006	↑
	Cyanobacteria	*norank_o__Gastranaerophilales*	0.020 ± 0.017	0.18 ± 0.091	0.04057	↑
	Firmicutes	*Christensenellaceae_R-7_group*	0.019 ± 0.0073	0.076 ± 0.034	0.04786	↑
	Firmicutes	*[Ruminococcus]_gauvreauii_group*	0	0.081 ± 0.032	0.01197	↑
	Firmicutes	*Anaerovorax*	0.011 ± 0.0038	0.062 ± 0.022	0.01706	↑
	Firmicutes	*Erysipelotrichaceae_UCG-003*	0.0013 ± 0.00023	0.043 ± 0.013	0.005516	↑
	Proteobacteria	*Desulfovibrio*	0.00012 ± 0.00009	0.013 ± 0.0057	0.006591	↑
	Firmicutes	*Dorea*	0.0038 ± 0.0018	0.022 ± 0.006	0.009755	↑
	Proteobacteria	*Escherichia*	0.00034 ± 0.00020	0.0015 ± 0.0011	0.03541	↑
	Firmicutes	*Gemella*	0	0.0091 ± 0.0011	0.000155	↑
	Firmicutes	*Ruminococcaceae_UCG-007*	0	0.0062 ± 0.0032	0.02915	↑
	Firmicutes	*Turicibacter*	0	0.057 ± 0.0098	0.007134	↑
	Verrucomicrobia	*Akkermansia*	0	0.00095 ± 0.00037	0.04312	
Day 118	Bacteroidetes	*norank_f__Bacteroidales_S24-7_group*	62.85 ± 13.96	35.30 ± 4.45	0.0312	↓
	Proteobacteria	*Parasutterella*	1.61 ± 0.30	0.056 ± 0.040	0.0008906	↓
	Firmicutes	*norank_f__Clostridiales_vadinBB60_group*	0.22 ± 0.058	0.059 ± 0.0076	0.009176	↓
	Actinobacteria	*Coriobacteriaceae_UCG-002*	0.089 ± 0.029	0.0027 ± 0.0047	0.007522	↓
	Tenericutes	*Ureaplasma*	0.0083 ± 0.0040	0	0.02443	↓
	Proteobacteria	*Desulfovibrio*	0.0071 ± 0.0033	0.015 ± 0.0021	0.007534	↑
	Firmicutes	*Ruminococcaceae_UCG-014*	0.51 ± 0.099	6.37 ± 0.98	0.001385	↑
	Firmicutes	*Unclassified_f__Lachnospiraceae*	2.20 ± 1.80	8.51 ± 1.64	0.01096	↑
	Firmicutes	*[Eubacterium]_coprostanoligenes_group*	0.020 ± 0.0030	3.64 ± 1.09	0.004533	↑
	Firmicutes	*Ruminococcus_1*	0.010 ± 0.0071	2.98 ± 1.47	0.02493	↑
	Bacteroidetes	*Alloprevotella*	0.066 ± 0.0068	1.36 ± 0.22	0.0006536	↑
	Firmicutes	*Anaerotruncus*	0.13 ± 0.060	0.83 ± 0.22	0.005963	↑
	Bacteroidetes	*Parabacteroides*	0.11 ± 0.033	0.60 ± 0.29	0.04347	↑
	Firmicutes	*Blautia*	0.050 ± 0.044	0.51 ± 0.25	0.03642	↑

	Firmicutes	*[Eubacterium]_nodatum_group*	0.085 ± 0.079	0.37 ± 0.093	0.0151	↑
	Firmicutes	*Lachnospiraceae_UCG-010*	0	0.38 ± 0.15	0.01046	↑
	Firmicutes	*[Eubacterium]_brachy_group*	0.036 ± 0.013	0.11 ± 0.034	0.02611	↑
	Proteobacteria	*Escherichia*	0.00014 ± 0.000098	0.0018 ± 0.00079	0.04519	↑
	Firmicutes	*Ruminococcus_2*	0	0.063 ± 0.08	0.04799	↑
	Firmicutes	*Dorea*	0	0.038 ± 0.013	0.008115	↑
	Firmicutes	*Gemella*	0	0.020 ± 0.0097	0.02624	↑
	Firmicutes	*Holdemania*	0	0.014 ± 0.0044	0.005278	↑
	Firmicutes	*Turicibacter*	0	0.078 ± 0.053	0.001348	↑
	Verrucomicrobia	*Akkermansia*	0	0.0052 ± 0.00013	0.008796	↑
Day 138	Firmicutes	*uncultured_f__Ruminococcaceae*	0.74 ± .055	0.37 ± 0.14	0.01395	↓
	Bacteroidetes	*Odoribacter*	0.89 ± 0.39	0.12 ± 0.063	0.02787	↓
	Firmicutes	*norank_f__Clostridiales_vadin BB60_group*	0.34 ± 0.16	0.049 ± 0.019	0.0372	↓
	Firmicutes	*Lactococcus*	0.27 ± 0.14	0.020 ± 0.016	0.04156	↓
	Bacteroidetes	*Bacteroides*	2.09 ± 1.01	8.37 ± 1.98	0.02891	↑
	Firmicutes	*Ruminococcaceae_UCG-014*	0.35 ± 0.075	3.81 ± 0.69	0.009553	↑
	Firmicutes	*Marvinbryantia*	0.27 ± 0.033	4.58 ± 0.79	0.003267	↑
	Proteobacteria	*Desulfovibrio*	0.0084 ± 0.0036	0.014 ± 0.0025	0.006249	↑
	Firmicutes	*Butyricimonas*	0	0.045 ± 0.019	0.01423	↑
	Verrucomicrobia	*Akkermansia*	0	0.0035 ± 0.00039	0.03547	↑
	Firmicutes	*Turicibacter*	0	0.0066 ± 0.0034	0.02583	↑
Day 178	Proteobacteria	*Parasutterella*	1.78 ± 0.86	0.13 ± 0.088	0.02929	↓
	Bacteroidetes	*Odoribacter*	0.56 ± 0.23	0.078 ± 0.043	0.02335	↓
	Bacteroidetes	*Unclassified_o__Bacteroidales*	0.48 ± 0.19	0.12 ± 0.047	0.0359	↓
	Firmicutes	*Bacillus*	0.059 ± 0.021	0.0084 ± 0.0034	0.01566	↓
	Proteobacteria	*Pseudomonas*	0.022 ± 0.0034	0.0027 ± 0.0046	0.004288	↓
	Firmicutes	*Carnobacterium*	0.016 ± 0.0081	0	0.02795	↓
	Firmicutes	*Exiguobacterium*	0.0079 ± 0.0046	0	0.04054	↓
	Firmicutes	*Ruminococcaceae_UCG-014*	0.83 ± 0.12	7.56± 1.51	0.008431	↑
	Firmicutes	*Butyrivibrio*	0.0091 ± 0.0059	4.32 ± 1.32	0.004812	↑
	Proteobacteria	*Desulfovibrio*	0.0067 ± 0.0021	0.011 ± 0.00081	0.001538	↑
	Firmicutes	*Quinella*	0.58 ± 0.054	2.01 ± 0.50	0.02788	↑
	Firmicutes	*Turicibacter*	0.0050 ± 0.0017	1.99 ± 0.50	0.002382	↑
	Cyanobacteria	*Microcystis*	0	0.51 ± 0.16	0.00506	↑
	Cyanobacteria	*norank_o__Gastranaerophilales*	0.029 ± 0.023	0.17 ± 0.071	0.02992	↑
	Firmicutes	*Anaerovorax*	0.0084 ± 0.011	0.13 ± 0.058	0.02597	↑
	Actinobacteria	*Gordonibacter*	0.016 ± 0.010	0.083 ± 0.038	0.03959	↑
	Firmicutes	*Family_XIII_AD3011_group*	0.013 ± 0.014	0.079 ± 0.011	0.00317	↑
	Proteobacteria	*Porphyrobacter*	0	0.052 ± 0.014	0.003051	↑
	Bacteroidetes	*Unclassified_p__Bacteroidetes*	0	0.034 ± 0.014	0.01311	↑
	Firmicutes	*Unclassified_f__Erysipelotrichaceae*	0.0027 ± 0.0024	0.018 ± 0.0070	0.02345	↑
	Proteobacteria	*Bilophila*	0.0027 ± 0.0024	0.010 ± 0.0021	0.01501	↑
	Verrocomicrobia	*Akkermansia*	0	0.00095 ± 0.00011	0.03528	↑
	Firmicutes	*Turicibacter*	0	0.020 ± 0.0093	0.02583	↑
